# Supplementary material for: Causal interplay between lactose intolerance and gut microbiota: a combined bidirectional Mendelian randomization and in vivo validation study
Source: Front Nutr. 2026 Jun 1;13:1803337. doi: 10.3389/fnut.2026.1803337 (PMC13265576; doi:10.3389/fnut.2026.1803337)
Supplement: Supplementary file 1 [file Data_Sheet_1.zip › supplementary materials/Forward/leave one out cross validation/ebi-a-GCST90027725.finngen_R12_E4_LACTONAS.pdf]

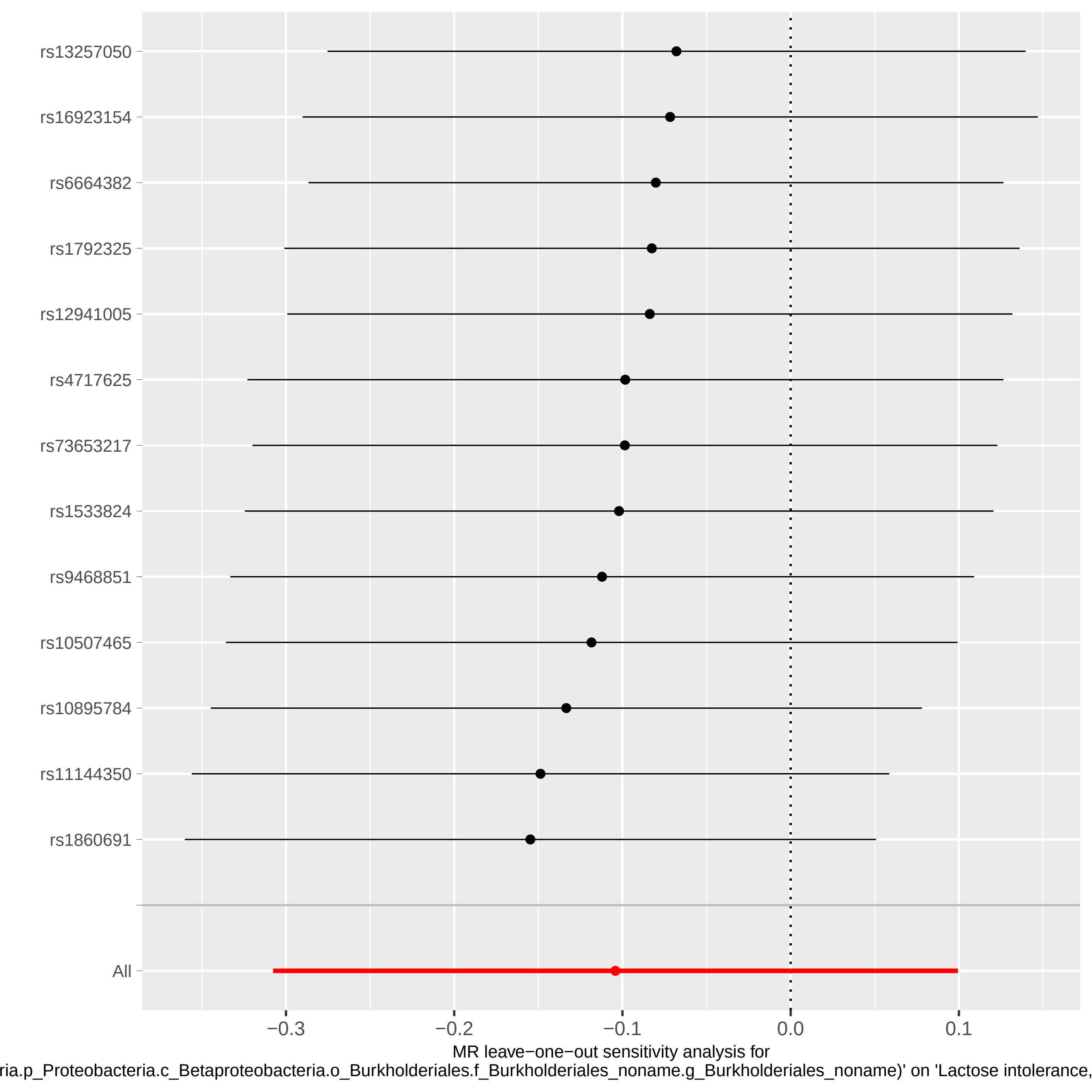
rs13257050
rs16923154
rs6664382
rs1792325
rs12941005
rs4717625
rs73653217
rs1533824
rs9468851
rs10507465
rs10895784
rs11144350
rs1860691
All
−0.3
−0.2
−0.1
0.0
0.1
MR leave−one−out sensitivity analysis for
ria.p_Proteobacteria.c_Betaproteobacteria.o_Burkholderiales.f_Burkholderiales_noname.g_Burkholderiales_noname)' on 'Lactose intolerance,
